# Function of Connexins in the Interaction between Glial and Vascular Cells in the Central Nervous System and Related Neurological Diseases

**DOI:** 10.1155/2018/6323901

**Published:** 2018-06-10

**Authors:** Yinan Zhao, Yanguo Xin, Zhiyi He, Wenyu Hu

**Affiliations:** ^1^Department of Neurology, The First Affiliated Hospital of China Medical University, Shenyang, Liaoning 110001, China; ^2^Department of Cardiology, West China Hospital of Sichuan University, Chengdu, Sichuan 610041, China; ^3^Department of Cardiology, The First Affiliated Hospital of China Medical University, Shenyang, Liaoning 110001, China

## Abstract

Neuronal signaling together with synapse activity in the central nervous system requires a precisely regulated microenvironment. Recently, the blood-brain barrier is considered as a “neuro-glia-vascular unit,” a structural and functional compound composed of capillary endothelial cells, glial cells, pericytes, and neurons, which plays a pivotal role in maintaining the balance of the microenvironment in and out of the brain. Tight junctions and adherens junctions, which function as barriers of the blood-brain barrier, are two well-known kinds in the endothelial cell junctions. In this review, we focus on the less-concerned contribution of gap junction proteins, connexins in blood-brain barrier integrity under physio-/pathology conditions. In the neuro-glia-vascular unit, connexins are expressed in the capillary endothelial cells and prominent in astrocyte endfeet around and associated with maturation and function of the blood-brain barrier through a unique signaling pathway and an interaction with tight junction proteins. Connexin hemichannels and connexin gap junction channels contribute to the physiological or pathological progress of the blood-brain barrier; in addition, the interaction with other cell-cell-adhesive proteins is also associated with the maintenance of the blood-brain barrier. Lastly, we explore the connexins and connexin channels involved in the blood-brain barrier in neurological diseases and any programme for drug discovery or delivery to target or avoid the blood-brain barrier.

## 1. Introduction

In the 1900s, the term “blood-brain barrier (BBB)” was firstly proposed to explain the reason that certain medicines failed to treat brain conditions [1]. The previous concept of BBB is a pure endothelial barrier of the brain, a physical barrier comprised of tight junctions (TJs) and adherens junctions (AJs) that limit paracellular diffusion of solutes between the blood and the brain [2, 3]. Currently, BBB is more likely to be considered as “a neuro-glia-vascular unit” [4, 5], a functional unit composed of capillar endothelial cells (ECs), glial cells, pericytes, and neurons and which interacts with local segments of blood vessels. Functionally, in healthy conditions, BBB serves as a higher selective barrier, protecting and separating the central nervous system (CNS) from toxic and damaging blood-borne compounds and peripheral neurotransmitter pools, protecting the synaptic and axonal signaling from ionic fluctuations, permitting the entry of required chemical molecules, and clearing larger molecules and brain metabolites, maintaining the balance of the brain microenvironment [6, 7]. In pathological conditions, brain-targeted diseases may be the origins, followed by vasculopathy, change of blood flow, and altered secretion of matrix molecules. Microglia activated and recruited by signals from astrocytes and neurons secrete more inflammatory cytokines, ultimately causing neuronal injury and synaptic dysfunction [5, 8–10].

According to previous studies, it is well known that the two kinds of vascular endothelial cell-cell junction, TJs and AJs, play key roles in the barrier function of BBB [2, 11]; nevertheless, very recently, researchers discovered a third category, gap junction (GC) channels, and the cross work with other cell-cell junctions in maintaining the barrier function [12]. Gap junction intercellular communication (GJIC) is formed by two hemichannels (HCs) located on the opposing cell membranes; each one is composed of six transmembrane proteins termed as connexins (Cxs) [13, 14]. Cx channels contribute to the intercellular communication between different cellular compartments. In addition, crapping around the capillary endothelial cells in CNS, dysfunction of vascular barriers caused by deficiency of Cxs on the membrane of astrocytes has been observed [15], indicating that Cx channels on astrocyte endfeet also play a pivotal role in the maintenance of function in BBB. However, it is still unclear how Cx channels work on astrocyte endfeet and contribute to the regulation of BBB. In addition, some believe that Cxs on the membrane of ECs rather than astrocytes are involved in the permeability status of BBB [16]. Accordingly, there are still debates about where and how Cxs regulate the barrier function and interact with other junctional proteins in CNS, hence the problem. This review focuses on the pivotal roles of Cxs and Cx channels in regulating the permeability of CNS/PNS barriers and related diseases caused by dysfunction of the “neuro-glia-vascular unit.”

## 2. BBB Structure

**Figure 1:**
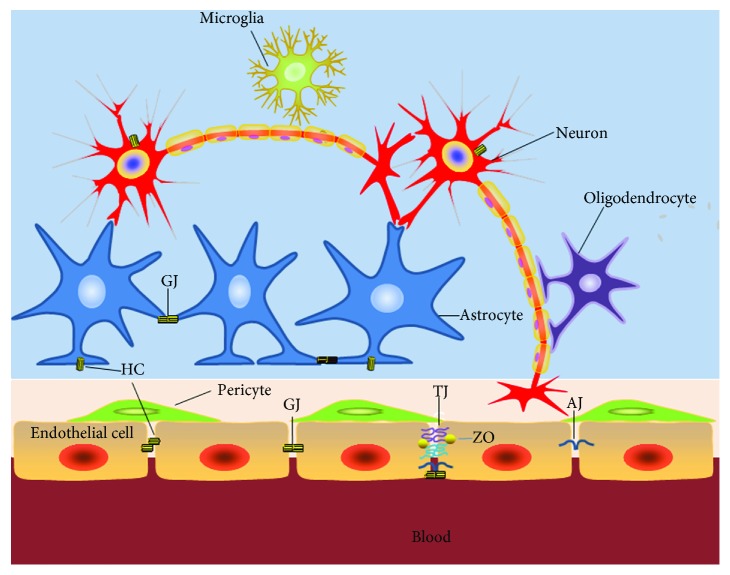
Connexin-based channels in BBB. A functional unit composed of capillary endothelial cells (ECs), glial cells, pericytes, neurons, and interaction with local segments of blood vessels.

BBB is considered as a functional unit, and glial cell is one of the important constituent structures, which forms a continuous membranous network around the vessels, where molecular signaling through pericytes and ECs is organized (Figure 1). This “highly selective permeability barrier” maintains brain homeostasis through permitting the entry of required nutrients and excluding potentially harmful compounds into the CNS [17, 18]. Thus, BBB plays a pivotal role in protecting the specific neuron function from biochemical attack in the systemic circulation.

**Figure 2:**
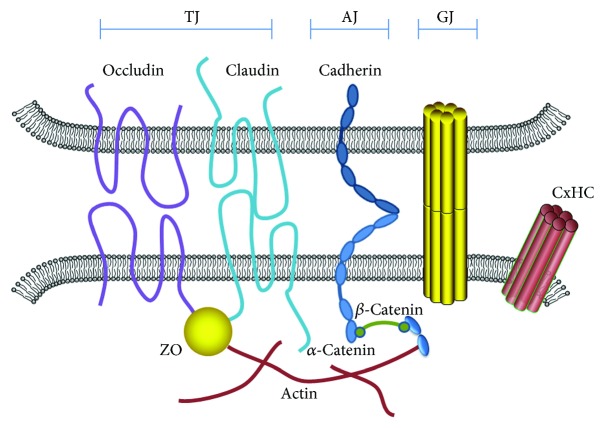
Cx channels form the intercellular junctional complex. TJ contains a complex of proteins spanning the intercellular cleft (occludin and claudin). The ZO protein family plays a role in anchoring the occludin and claudin to the cytoskeleton via interaction with actin. The AJ protein, cadherin, spans the intercellular cleft and provides structural support. Transmembrane Cx proteins constitute a third partner in the intercellular junctional complex. GJ is formed by two HCs located on the opposing cell membranes, which are composed of six transmembrane Cx proteins.

Capillary ECs, another constituent structure of BBB, supply a junctional complex comprising of AJs and TJs and control paracellular diffusion of solute between the blood and the brain [2, 4, 11, 19] (Figure 2). TJs contain a complex of proteins spanning the intercellular cleft (occludin and claudin) and junctional adhesion molecules [2, 20]. The zona occludin protein family (ZO-1, ZO-2, and ZO-3) plays a key role in anchoring the occludin and claudin to the cytoskeleton via interaction with actin [21]. The AJ protein, cadherin (*α*, *β*, and *γ*), spans the intercellular cleft and provides structural support [2]. Recently, more focus is on the significant role of Cx proteins in the junctional complex [22, 23]. Unlike TJ and AJ proteins, Cx proteins do not form a tight seal between the connecting cytoplasm and adjacent cells, despite adhesive properties indispensable for the adjacent cells.

## 3. Cxs in Glial Cells and Neurons

**Figure 3:**
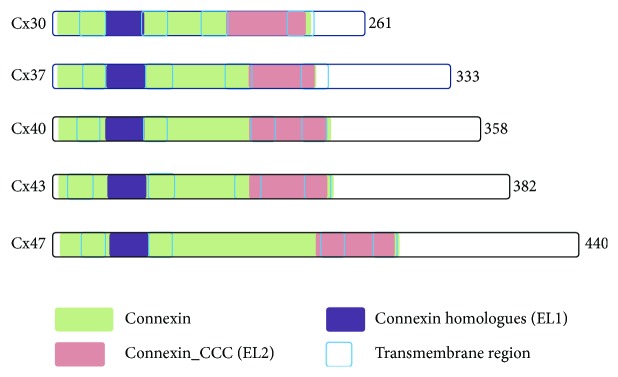
Schematic representation of connexins. Each Cx is a four-pass transmembrane (red frame) protein comprising two extracellular loops (EL1, purple region; EL2, green region) and a C-terminal cytoplasmic tail. The domains and motifs of connexins are depicted according to the size.

GJs constitute connected exchange channels between adjacent cells for the interchange of cytoplasm components, small molecules, ions, and so on, and maintain metabolic cooperation [24–26], completeness of intercellular signaling pathways, liquid buffer, and electric coupling [27, 28]. GJs are formed by two HCs located on the opposing cell membranes (Figure 2), which are composed of six transmembrane proteins termed as Cxs. The Cx superfamily is comprised of 21 isoforms in humans and 20 isoforms in mice in a tissue-specific expression pattern and named according to their molecular weight (Figure 3) [29, 30].

Eleven kinds of Cxs have been detected in the rodent brain [31]; Cx30 and Cx43 are two major categories expressed in astrocytes. The expression of astrocytic Cxs varies by brain region and stage of development [31]. For instance, the time pattern of the postnatal expression of Cx30 (from P12) and Cx43 (from P2) in astrocyte endfeet correlates with the maturation of the BBB [15, 32]. White matter astrocytes express minimal or no Cx30 [33]. In contrast, expression of Cx30 is higher compared with that in white matter, even more than Cx43, in the thalamus [34]. Lack of Cx43 leads to the reduction of coupling and absence of both Cx43 and Cx30 abolishment of interastrocyte coupling [35–37], indicating that Cx43 and Cx30 are the major components of a direct intercellular communication between astrocytes. Cx36, together with a site-specific expression of Cx45 and Cx57, is the principal connexin expressed in neurons. Cx36 was firstly reported to be expressed specifically in mammalian neurons [38]. The expression of Cx36 is sharply reduced during the course of postnatal maturation and is sparsely expressed in the neocortex; however, it remains enriched in subsets of interneurons in the hippocampus, olfactory bulb, and thalamus [38–40]. There was evidence indicating the absence of GJs between neurons and glial cells in the mammalian CNS [31, 41, 42]; however, both electrical and dye coupling between Bergmann glial cells and Purkinje neurons has been observed in adult rats [43].

## 4. Cxs in Different Regions of Vascular Tree (Arteries, Arterioles/Venous System, and Lymphatic System)

The expression regions of Cx40, Cx37, and Cx43 are mainly restricted on the cytomembrane of vascular ECs [44]. Cx37 and Cx40 are widely expressed in arteries and arterioles, while Cx43 in the regions is consistent with turbulent blood flow [45–47]. Cx37 expression is prominent in the straight portion of carotid arteries, but reduced from the carotid bifurcation [48]. Cx43 is robustly expressed at branching vessels; turbulent shear stress caused by disturbed blood flow makes the endothelium of these small vessels prone to developing atherosclerotic lesions [46, 49].

Very limited studies focused on the expression pattern of Cxs in the venous/lymphatic system. Cxs expression (Cx37, Cx40, and Cx43) in the venous system also exhibited the same manner as in the arterial systems, which is a regional variation [50].

Kanady et al. observed Cx37, Cx43, and Cx47, especially the first two which exhibited a regional and dynamic variation of expression manner in mice. Interestingly, coexpression of Cx47 and Cx43 was observed in the region upstream of the adult valve. A knockout mouse model showed that Cx37 and Cx43 but not Cx47 affected lymphatic morphology [51–53].

Microvasculature is the region where the exchange of nutrients and materials between tissue and blood takes place. As mentioned above, the expression of Cx43 is higher in microvasculature compared with that in macrovasculature [54], indicating that Cx43 is a major regulator of vascular permeability. Upregulation of Cx43 in ECs, associated with the vascular hyperpermeability, has been verified to be consistent with the above conclusion [54–57].

## 5. Function of Endothelial Cxs in BBB

Endothelial cells individually or jointly express Cx37, Cx40, and Cx43, in a vessel type-dependent manner as mentioned above. Several evidences point to the possible contribution of endothelial Cxs in maintaining the permeability status of the BBB. Cx40 and Cx43 are associated with TJ proteins (occludin and claudin-5) in porcine brain ECs through cytoplasmic scaffold proteins, such as ZO-1. GJ blocker 18 *β*-glycyrrhetinic acid and oleamide did weaken the barrier function of TJs, but did not influence the expression or distribution of Cxs or TJ proteins in porcine BBB ECs [12] (Table 1). Damaged BBB permeability was considered to be caused by loss of function of GJIC and TJs without morphological change; however, the mechanism needs further research. In contrast with pathological conditions, the expression of Cx43 in the endothelial wall of healthy blood vessels appears low and increases after inflammation occurred, especially in the endothelial cells of smaller blood vessels including those of the BBB [55, 58]. Cx43 antisense treatment further reduces vascular permeability and invasion of neutrophils in spinal cord injury (SCI) [55] (Table 2). Very recently, increased expression of Cx43 and permeability of BBB were observed in familial cerebral cavernous malformation type III (FCCM3) lesions of mouse model brains [59], partly consistent with the observation in *ccm3* knockdown brain microvascular ECs, which was accompanied by dislocation of ZO-1, limitation to formation, and transinteraction of TJs *in vitro*. Gap27, a Cx43 GJ inhibitor, rescued CCM3KD hyperpermeability through inhibiting Cx43 GJs, restoring ZO-1 to TJ structures, and reducing plaque accumulation in Cx43 GJs, indicating that Cx43 GJs are increased in Fccm3 and regulate barrier permeability in a TJ-dependent manner [59] (Table 1). It is unclear by what mechanism CCM3 regulates Cx43 and whether Cx43 is exclusive to participate in the formation of TJs.

## 6. Involvement of Glial and Neuronal Connexins and BBB Function

In the nervous system, astrocytic Cx is unique and contributes pivotally to brain metabolism and processing [60]. Cx30 and Cx43 are two major Cx proteins expressed by astrocytes and that form Cx-based channels to mediate intercellular signaling, especially that involving Ca^2+^ and maintaining the BBB integrity [15, 32]. A knockout model was induced to clarify the role of astroglial Cxs in the morphology and function of BBB. The combination of global *Cx30* and astrocyte-specific *Cx43* deletion (conditional knockout from glial fibrillary acidic protein-expressing cells) downregulates aquapoorin 4 (AQP4), which is located in astrocytes throughout the CNS in the brain, spinal cord, and optic nerve and is particularly concentrated in foot processes adjacent to microvessels at BBB [61] (Table 2). In contrast, TJ proteins, occludin, and ZO-1 show no difference in expression or morphology, compared with the WT mice [15]. Consequently, double knockout of astrocytic *Cx43* and *Cx30* leads to systemic astrocyte endfeet swell and increased BBB permeability with the increase in vascular and shear stress [15]. However, absence of Cx30 did not affect organization or permeability of BBB according to a further analysis of the separate role of Cx30 [62] (Table 2). Absence of astroglial Cx43 compromises the ability of the brain to maintain immune quiescence [63]; recruitment of T cells, B cells, macrophages, and neutrophils; enhanced antigen presentation; and autoimmune reaction [64–66] (Table 2). Immune cell recruitment is not the consequence of BBB breakdown in *Cx43-KO* mouse brain, despite progressively weakened BBB permeability under shear and pressure [64]. Loss of immune-quiescent, immune-competent cells abnormally crossing the BBB and developing autoimmune mechanisms could be harmful as suggested in pathological conditions linked to a decreased expression of Cx43 in multiple sclerosis (MS) and neuromyelitis optica (NMO) patients [67, 68]. Deficiency of Cx43 nonchannel function is associated with migration impairment of neurons through adhesive interaction with radial fibers during embryogenesis [69, 70] (Table 2). However, the mechanism by which knockdown of Cx43 leads to the migration of immune-competent cells abnormally crossing the BBB and the Cx-channel-dependent or nondependent function important to mediate the migration process needs to be clarified further.

In an ultrasound-induced BBB opening Wistar rat model [71], reorganization of neuronal Cx36-containing GJs with an increase in gap-junctional plaque size was found, similar to the results in another study [72]; neuronal Cx36 interacts with a ZO-1 PDZ domain, the same as in astrocytic Cx43, thus indicating that ultrasound-induced disruption of ZO-1 might lead to an increase in Cx36 plaque size. However, in the same BBB opening model [71], changes in Cx36 plaque size were not as striking as in astrocytic Cx43 plaques, suggesting the key role of astrocytes for the maintenance of homeostasis of BBB.

## 7. The Roles of Cx Channels in BBB Function

Several factors, such as inflammatory cytokines and growth factors, contribute to the regulation of Cx-HC and GJ channels in physiological or pathological progress and modulatory mechanism of BBB. Accumulating evidence indicated that Ca^2+^ transients in astrocyte endfeet may induce dilation and constrictions in the adjacent arterioles and may further precede changes in blood flow and disturbance of the healthy brain function [73, 74]. One research showed an increased BBB and bovine brain capillary EC permeability through triggering calcium oscillations involved in Cx-HC opening, indicating that HCs may play a vital role in the process of calcium signaling and propagation of ICW [57]. Divergent functional properties of Cx43 channels have been set forth in inflammatory conditions. Retamal et al. found that the proinflammatory cytokines IL-1*β* and TNF-*α* reduced the intercellular communication via Cx43 GJIC, whereas it increased the cellular exchange with the extracellular milieu via Cx43 HCs [75]. Inhibition of GJIC that is observed under the condition of inflammation indicates that the increased membrane permeability may be attributable to active HCs [75, 76]. On the other hand, the effect of proinflammatory cytokines enhanced the uptake and reduced the intercellular diffusion of glucose, which might explain the change of metabolic status in astrocytes under inflammatory conditions. Accordingly, such opposite regulation effects of Cx channels may impact glucose trafficking and modify the metabolic status of astrocytes involved in brain inflammation [75]. Connexin channels provide a target to manipulate brain endothelial calcium dynamics and BBB permeability [57]. Bradykinin, a typical inflammatory messenger, increases BBB and rat brain endothelial (RBE4) cell permeability through triggering calcium oscillations involved in Cx-HC opening. Gap27, a GJ mimetic peptide identical to part of the second extracellular loop of Cx37 and Cx43 structurally, reduced intracellular oscillations in RBE4 cells in response to bradykinin, in accordance with the Cx37/Cx43-targeted siRNA [57] (Table 1). Another group indicated that intercellular Ca^2+^ waves (ICWs) increased the endothelial permeability by lowering the extracellular Ca^2+^. The endothelial permeability increase was furthermore inhibited by Gap27, which also blocked the ICWs, and with inhibition of protein kinase, Ca^2+^/calmodulin-dependent kinase II, and actomyosin contraction. In conclusion, ICWs significantly increase endothelial permeability and the Cxs underlying Ca^2+^ wave propagation form a target to limit BBB alteration [77] (Table 1).

GJs mimetic peptides, Gap26 and Gap27, which specifically target Cx channels, are widely used to close the HCs and, in certain conditions, gap junction communication [78]. ATP is the best known signaling factor linked to Cx-HC activity and released from astrocytes directly through Cx43-HCs [79]. Gap26 binds to the first extracellular loop of Cx43 and blocks Cx43-HCs in ECs, demonstrating that intracellular ATP is released through Cx-HCs in a Cx-dependent manner, which contributes to the intercellular propagation of calcium signals, thereby affecting the postulated spatial spread of BBB opening [80, 81] (Table 1). Nevertheless, one *in vitro* study showed that Gap26 has no effect on the elevated baseline ATP from rat brain ECs treated by TNF-*α*, suggesting that ATP release is non-Cx-dependent under proinflammatory cytokine-stimulated conditions [82] (Table 1). Gap27 targets the second extracellular loop of Cx37 and Cx43, and exposure to Gap27 upon short time (minutes) inhibits Cx-HC-mediated ATP release and dye uptake [32, 81, 83], while longer exposure duration (hours) blocks junctional coupling, as well [81, 84]. Gap27 limits BBB permeability induced by bradykinin *in vivo* through reducing intracellular calcium oscillations without modifications of TJs and cytoskeletal proteins such as occludin or ZO-1 [57] (Table 2). Some problems remain to be solved. First, some GJ mimetic peptides are widely used to inhibit the HC and GJ channels; however, what role GJ channels and HCs play in glial endfeet and how they contribute to regulation of BBB permeability need to be explained in detail. Secondly, Wnt, together with the Sonic hedgehog (Shh) signaling pathway, regulates the maturation of the BBB during development [85–87]; however, which signaling molecules that passed through Cx channels contribute to astrocyte endfeet structure adhesion and affect the integrity of BBB also needs to be clarified.

## 8. Cxs and Cx Channels in Nervous System Diseases

### 8.1. Multiple Sclerosis

Downregulation of oligodendrocytic Cx32, Cx47, and astrocytic Cx43 had been identified in the active lesions of CNS in MS patients and EAE mice [88–90]. Lutz et al. reported that deletion of both *Cx30* and *Cx43* in the EAE model did not aggravate clinical signs or pathological progress; moreover, no difference was observed in BBB permeability [91], indicating that absence of astrocytic Cx43 and Cx30 does not contribute to the pathology progress of the nerve tissue or further weaken BBB intensity under inflammatory condition (Table 3).

### 8.2. Neuropathic Pain

Inflammatory-mediated pain alters in TJs; ZO-1 expression was significantly increased, increasing the permeability of the BBB of the carrageenan-induced inflammatory pain rat model [92] (Table 3). An in vivo evidence suggests that carrageenan-induced spinal IL-1*β* inhibits astrocyte activation during the early phase of inflammation through the suppression of Cx43 expression [93]. Cx43 has been reported to induce chemokine CXCL1 (keratinocyte-derived cytokine) release to contribute to neuropathic pain. TNF-*α* activated Cx43-HCs, rather than GJIC, to release CXCL1 [94], which could be blocked by Gap26, Gap27, and *Cx43*-siRNA in cultured astrocytes from the cortex and spinal cord of the SCI mouse model, a mouse model for chronic neuropathic pain (Table 1). Cx43 is increased in cervical level 6 to 8 dorsal root ganglia, and specific peptide inhibitors of Cx43 ameliorated established tactile allodynia after severe SCI. Notably, SCI-induced mechanical allodynia was prevented in *Cx43/Cx30* double-knockout mice, highlighting the importance of glial intercommunication in chronic neuropathic pain [95, 96].

### 8.3. Stroke

*Cx43* deficiency not only creates intercellular communication reduction and weakened barrier function of BBB but also exacerbates ischemic injury [15, 97, 98] (Table 3). After ischemia-reperfusion, endothelial Cx expression is upregulated and Cx-HCs open [57, 99, 100], leading to glutamate excitotoxicity and inflammatory mediator release including nitric oxide [101] and further leading to EC edema and rupture [56, 102], astrocyte activity, T cell and monocyte migration, and increased inflammatory reaction, ultimately inducing tissue damage [103–105]. Inhibition of astrocytic GJs by octanol, for instance, could restrict the flow of neurotoxins, which exacerbate neuronal damage of cerebral ischemia [106]. In addition, Cx43 mimetic peptide, a Cx43 inhibitor, exhibited protective effects after cerebral and retinal ischemia [58, 107], which proves the point in reverse.

### 8.4. Alzheimer's Disease (AD)

An age-related decline in BBB integrity correlates with the loss of sex steroids and increase in gonadotropins with menopause [108]. Ovarietomy of young female mice induces an increased expression and dislocation of Cx43 in ECs accompanied by a rise in the permeability of the BBB without change of TJ protein, ZO-1 [108] (Table 3). It implicates Cx43 in regulating changes in BBB permeability and serum gonadotropins in the cerebral pathophysiology of neurodegenerative diseases, such as AD. One study reported that amyloid *β* (A*β*) peptide, a self-aggregating 40–42-amino-acid protein that is crucially involved in AD as the main component of the amyloid plaques, did harm to neurons resulting in their dysfunction and death [109], contributing to AD pathology through dramatically enhancing GJC/HC-mediated Ca^2+^ wave propagation in astrocytes [110] (Table 1). Recently, several studies [111–113] indicated that expression of astroglial Cxs is increased at amyloid plaques and that there is an increase in HC activity in reactive astrocytes that should certainly have an impact on gliovascular interaction. Until recently, INI-0602, a putative HC blocker, was verified to inhibit LPS-induced glutamate release from microglia *in vivo* and improve memory deficits in double transgenic mice expressing human amyloid precursor protein with K595N and M596L mutations and presenilin 1 with A264E mutation as an AD mouse model [114] (Table 2). An important next step underlying *Cx32* conditional knockout in microglia will be needed to demonstrate the unique regulation of microglial HCs in the progress of AD.

### 8.5. Epilepsy

TGF-*β* signaling is a key trigger of albumin-induced epileptogenesis, following BBB breakdown, which can be inhibited by the TGF-*β* pathway blocker and can manifest the TGF-*β* pathway as a novel therapeutic target for preventing injury-related epileptogenesis [115, 116]. Downregulation of GJ proteins Cx43 and Cx30, inwardly rectifying K^+^ channels (Kir4.1), the glial excitatory amino acid transporters (EAAT1 and EAAT2), and dislocation of aquaporins (AQP4), represents astrocyte activation induced by BBB disruption, as well as by focal application of albumin or TGF-*β*. These data indicate that activation of astrocytes is a critical step in epileptogenesis preceding alteration in neuronal function [115, 117]. Although many of those signaling cascades are altered in activated astrocytes, very little is known about neurovascular coupling in epilepsy. In the immature rat brain, GJIC is involved in rhythm genesis and synchronization of cortical activity and may enhance the epileptogenicity of the 4-aminopyridine- (4-AP-) induced epilepsy model [118]. In addition, repeated seizures can induce changes in different Cx gene (Cx26, 32, and 43) expression during the postnatal development, especially gradually elevated expression of Cx43 and GJIC remodeling [118] (Table 3). Assuming a selective inhibition of a Cx43-dependent process by the mimetic peptides and preferential localization of this Cx isoform in astrocytes suggests that in developing hippocampal networks, the generation and initiation of spontaneous recurrent seizure-like activity may depend in large part upon the opening of glial GJs [119] (Table 1).

### 8.6. NeuroAIDS

The main cells infected with HIV within the CNS are macrophages, microglia, and a small fraction of astrocytes [120–122]. Both *in vitro* and *in vivo* evidence uncovered a novel mechanism of bystander BBB toxicity mediated by low numbers of HIV-infected astrocytes and amplified by GJs [123] (Table 3). Few HIV-infected astrocytes compromise BBB integrity through blocking GJ channels, indicating that HIV affects the CNS by a GJ-dependent manner [123].

## 9. Discussion

Homeostasis of metabolites and ions is strictly regulated by the barrier between blood and nerve tissue. The BBB shields the direct contact between systemic circulation and the brain, as well as the spinal cord and peripheral nerve. TJs and AJs, two pivotal junctional complexes physically located in the ECs, are considered as the main structural components of the barriers. In this review, we introduce a third junctional component, Cx proteins, as an important player in the interactions with other junctional complexes that occur at the neuro-glia-vascular interface. In a cerebral microenvironment, Cx-forming channels of both astrocytes and ECs are critical in the control of BBB function. Interestingly, GJ channels and HC channels of BBB appear to play opposite roles in the regulation of BBB permeability. GJIC contributes to maintaining BBB integrity, while HCs are associated with ATP signaling release and necessary to generate the Ca^2+^ oscillations linked to BBB disruption induced by proinflammatory signals. A cluster of papers have provided strong evidence in favor of a Cx channel-dependent manner contributing to the regulation of BBB function; however, few researchers focus on a Cx channel-independent manner, adhesive contact, and gene expression, for instance [16, 124, 125]. It remains to be demonstrated how nonchannel Cxs interact with other cell-cell-adhesive proteins to maintain the barrier. Cxs have been considered as novel therapy targets for neurologic diseases in connection with BBB dysfunctions. Astrocytic and endothelial Cx channels are vital to BBB permeability change caused by neurologic pathology, especially inflammation. Microglial cells also play an important role in activating immune cells; however, the role of Cxs in the intercellular cross talk between BBB endothelial cells and microglia, and microglia in the BBB development in physiological or pathologic conditions, remains unclear. More studies should be focused on the mechanism of microglia-endothelial Cx channels in BBB permeability regulation.

## Figures and Tables

**Table 1:** Connexin inhibitors link to the BBB permeability *in vitro*.

Cell type	Stimulus	Treatment	Main outcome	Reference
Porcine BBB ECs		18*β*-Glycyrrhetinic acid and oleamide	Inhibits the barrier function of TJs and GJIC without morphological change	[12]
Mouse brain CCM3KD ECs		Gap27	Inhibits BBB hyperpermeability through blocking Cx43 GJs, restoring ZO-1 to TJ structures	[59]
Rat brain RBE4 cells	Bradykinin	Gap27, *Cx37*-siRNA, and *Cx43*-siRNA	Inhibits endothelial hyperpermeability through reducing intracellular calcium oscillations	[57]
Rat brain RBE4 cells	A reduction in extracellular Ca^2+^ concentration	Gap27	Inhibits endothelial hyperpermeability through blocking the intercellular Ca^2+^ waves and repressing PKC, CaMKII, and actomyosin contraction	[77]
Rat brain (RBE4, GP8 cells)	TNF-*α*	Gap26	No effect on the elevated baseline ATP release from rat brain endothelial cells treated by TNF-*α*	[82]
Rat brain GP8 cells		Gap26 and Gap27	Inhibits intracellular ATP release through blocking Cx43-HCs which contribute to the intercellular propagation of calcium signals	[81]
Organotypic hippocampal slices from 7-day-old Wistar rats	Albumin	Carbenoxolone, Gap27, and SLS peptide	In developing hippocampal networks, the generation and initiation of spontaneous recurrent seizure-like activity depend on the opening of glial GJs	[119]
Astrocytes from the cortex and spinal cord of the SCI mouse model	TNF-*α*	Gap26, Gap27, and *Cx43*-siRNA	TNF-*α* activated Cx43-HCs, rather than GJIC, to release CXCL1 which contribute to neuropathic pain	[94]
Astrocytes from cortex of Sprague-Dawley rat	A*β*-peptide	Octanol (an uncoupler of gap junctions)	Functional GJs are not required for calcium-wave propagation; they play a role in the enhancement of calcium waves induced by A*β*.	[110]

ECs: endothelial cells; SCI: spinal cord injury; TJs: tight junctions; GJs: gap junctions; ZO: zona occludin; PKC: protein kinase C; CaMKII: Ca^2+^/calmodulin-dependent kinase II; HCs: hemichannels; GJIC: gap junction intercellular communication; A*β*: amyloid *β*.

**Table 2:** Connexin inhibitors link to the BBB permeability *in vivo*.

	Disease model	Interference	Main outcome	Reference
Spinal cord	SCI	Cx43-asODN	Reduce vascular permeability and invasion of neutrophils in spinal cord injury	[55]
BBB	Neuroinflammation	Gap27	Gap27 coadministered together with bradykinin reduced leakage of reporter dye from the vascular lumen into the tissue	[57]
BBB		Global *Cx30* and astrocytic-specific *Cx43* deletion	Systemic astrocyte endfeet swell and increased BBB permeability with the increase in vascular and shear stress	[61]
BBB		Global *Cx30* deletion	Not affecting organization or permeability of BBB	[62]
BBB		Astrocytic-specific *Cx43* deletion	Compromises the ability of the brain to maintain immune quiescence and recruitment of immune cells without BBB breakdown	[63–66]
Embryo		*Cx43* deletion	Migration impairment of neurons through adhesive interaction with radial fibers	[69, 70]
Brain	AD	INI-1602 (microglia HC blocker)	Improve memory deficits	[114]

SCI: spinal cord injury; HCs: hemichannels; BBB: brain-blood barrier.

**Table 3:** Cxs/Cx-channels in BBB in CNS pathology.

CNS pathology	Cxs/Cx channel remodeling	BBB dysfunction	Reference
MOG-induced EAE mouse model	Deletion of both *Cx30* and *Cx43*	None	[126]
Carrageenan-induced inflammatory pain rat model	Not mentioned	Increased expression of ZO-1 in TJs and permeability of the BBB	[92]
MCAO mouse model	*Cx43 * ^+/−^, GJIC reduction	Weakened barrier function of BBB and exacerbated ischemic injury	[98]
Age-related neurodegenerative disease mouse model	Increased expression and relocalization of Cx43 in ECs	An increase in the permeability of the BBB without change of ZO-1	[108]
4-AP-induced epilepsy rat model	Gradually elevated expression of Cx43 and GJIC remodeling	Not mentioned	[118]
HIV-infected neuroAIDS macaque model	HIV amplified by GJs	Compromised BBB integrity	[123]

Cx: connexin; TJs: tight junctions; GJs: gap junctions; ZO: Zona occludin; GJIC: gap junction intercellular communication; BBB: brain-blood barrier; 4-AP: 4-aminopyridine.
